# A Randomized controlled trial of the effect of yoga and peer support on glycaemic outcomes in women with type 2 diabetes mellitus: a feasibility study

**DOI:** 10.1186/s12906-017-1574-x

**Published:** 2017-02-07

**Authors:** Aswathy Sreedevi, Unnikrishnan Ambika Gopalakrishnan, Sundaram Karimassery Ramaiyer, Leelamoni Kamalamma

**Affiliations:** 1 Community Medicine, Amrita Institute of Medical Sciences, Amrita Vishwa Vidyapeetham, Kochi, Kerala India; 2 Dept of Endocrinology, Amrita Institute of Medical Sciences, Amrita Vishwa Vidyapeetham, Kochi, Kerala India; 3 Biostatistics, Amrita Institute of Medical Sciences, Amrita Vishwa Vidyapeetham, Kochi, Kerala India; 4 Community Medicine, Amrita Institute of Medical Sciences, Amrita Vishwa Vidyapeetham, Kochi, Kerala India

**Keywords:** RCT, Type 2 Diabetes, Women, Glycaemic outcomes, Yoga, Peer support

## Abstract

**Background:**

Type two diabetes is a complex and demanding chronic disease and its impact in a state (Kerala) which leads India in terms of the number of people with Diabetes is profound. Though the male to female ratio among the people with diabetes is roughly equal, women are uniquely and more severely affected. Management of type two Diabetes requires considerable dexterity on the part of the patient to manage drugs, diet and exercise. Therefore, in a low middle-income country like India it is necessary to look at low cost interventions that can empower the patient and build on available resources to help manage diabetes. Hence, we studied the feasibility and effect of two low cost interventions; yoga and peer support on glycaemic and other outcomes among women with type two diabetes.

**Methods:**

An open label parallel three armed randomized control trial was conducted among 124 recruited women with Diabetes for three months. Block randomization with a block length of six was carried out with each group having at least 41 women. In the Yoga arm, sessions by an instructor, consisting of a group of postures coordinated with breathing were conducted for an hour, two days a week. In the peer support arm each peer mentor after training visited 13–14 women with diabetes every week followed by a phone call. The meeting was about applying disease management or prevention plans in daily life.

**Results:**

There was a trend in decline of fasting plasma glucose in the peer and yoga group and of glycosylated haemoglobin (HbA1c) in the yoga group only, though not significant. A significant decrease was observed in diastolic blood pressure and hip circumference in the yoga group. The process indicated that most (80%) of the women in the yoga group attended classes regularly and 90% of the women in the peer group reported that peer mentoring was useful.

**Conclusion:**

The effect of yoga and peer support on glycaemic outcomes was incremental. Longer term studies are necessary to ascertain the benefits shown by this feasibility study.

**Trial registration:**

CTRI/2011/12/002227 dated 14/12/2011.

## Background

There are about 66 million people in India with type two Diabetes according to the 2014 International Diabetes Federation (IDF) [1] reports with a prevalence of 8.3% [2, 3]. Kerala, a southern state in India where the study was carried out leads the country with a prevalence of 20% [4] and is a harbinger of what will happen in future to the rest of India [5]. Higher rates of cardio-metabolic risk factors such as central adiposity and high insulin resistance in the Indian and south Asian population presents at younger ages [6]. Policy makers in countries with limited resources are required to prioritize amongst the list of important public health problems on the basis of disease burden, cost effectiveness and equity [7]. In addition, the health care system in India has also evolved around the concept of acute care for infectious diseases and the system performs best when addressing patients with episodic and urgent concerns [8].

While the ratio of men-to-women afflicted with diabetes is roughly equal, women are uniquely and often more severely, affected by complications of diabetes [9, 10]. The prevalence of overweight, abdominal obesity are also high among women and they also suffer from related complications like elevated lipids and blood sugar levels [11]. Type two Diabetes is a complex and demanding chronic disease requiring considerable dexterity on the part of the patient to manage drugs, diet and exercise. Therefore, it is necessary to look at low cost interventions that can empower the patient and build on available resources. Yoga and peer support can be considered to be two such interventions which can empower the patient to institute behavior change and adhere to the complex and demanding nature of this chronic disease.

Yoga, an ancient Indian psychological, physical and spiritual exercise regimen has been studied for control of symptoms and complications associated with type two Diabetes Mellitus (DM). Jain et al. has suggested that Yoga can be an adjuvant in the management of type two DM [12]. Systematic reviews conclude that according to current evidence Yoga may be efficacious but a well-designed, randomized control trial is necessary to prove its efficacy [13].

The second intervention, Peer support has empowerment as the theoretical basis. It has been found that experiential support provided by lay people is useful in the management of diabetes mellitus, including glycaemic control [14]. Peer support has been defined as the support from an individual with experiential knowledge based on a sharing of similar life experiences or prevention plans in daily life [15].

Thus, this study aimed at studying the effect of yoga and peer support on glycaemic outcomes, pharmacological adherence and anthropometric measures.

## Methods

The estimated sample size based on earlier study [16] on the study variables-Fasting plasma glucose, Glycosylated haemoglobin (HbA1c) and Quality of life at 95% confidence and 80% power varied from 5–40 with an anticipated fall in Fasting plasma glucose (FPG) of 32 mgm/dl and a fall in HbA1c of at least 0.5%. The maximum sample size being 40 the required sample size for the study is 40 for each group totaling 120 for the open label three armed randomized trial.

### Study population and Recruitment

**Fig 1:**
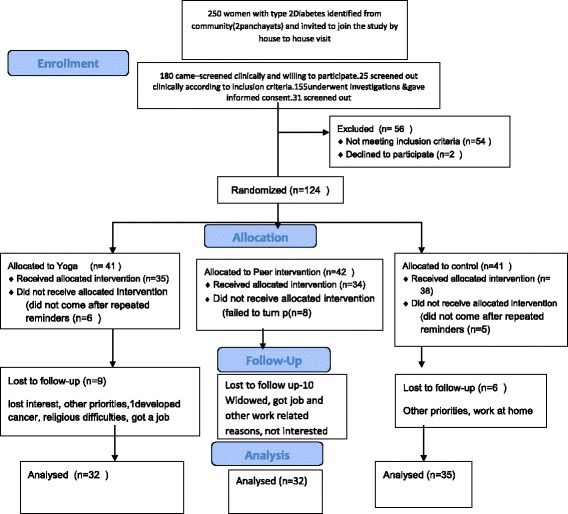
Flow diagram of enrollment and follow up

The study was conducted in a rural area of Kerala, India at a rural health training centre. The interventions; yoga and peer support were administered for a period of three months. From a population of 19, 000 residing in the self administration-unit (panchayat), 1042 cases of type two diabetes were listed on the basis of a baseline survey in the area (Hospital records, Amrita Community Health Training Centre, Njarackal,Ernakulam district, Kerala,). The women from this list in the 30–65 age groups were contacted and the first 250 women with diabetes were identified and invited to participate in the study. The age 30 was chosen as it was less likely for women less than 30 years to be affected by type two diabetes. The upper age limit was 65 years as they constitute the elderly age group and are more likely to have mobility issues related to osteoarthritis. After clinical screening, willingness and applying the inclusion and exclusion criteria 124 women were enrolled in the study and randomized into three groups (Fig 1).

#### Inclusion Criteria

Women with type two DM diagnosed within the last 8 years and between 30–65 years of age, HbA1c between 7–10% were included.

#### Exclusion Criteria

Those already practicing Yoga, Meditation, women with Chronic Alcohol consumption and those with known diabetes complications, pregnant and lactating women, women with diabetes on alternate system of medicine treatment, Body Mass Index (BMI) > 35 kg/m2 and those with serious medical and psychiatric conditions were excluded.

After obtaining written informed consent, the women underwent a baseline assessment by a physician. Those on pharmacologic or non pharmacologic treatment continued with the same. All the study participants were given an education on diet and were advised exercise for at least 10 min per day.

### Randomization

The participants were randomized by block randomization of fixed block length of 6. All possible permutations of the three arms A, B, C were listed. A list of independent identically distributed random numbers were generated and chosen. Each number in this list was then replaced by the block. The random allocation sequence was generated by a person not connected with the study at the main hospital and handed over to the research assistant at the health centre in numbered opaque sealed envelopes. The allocation ratio was approximately 1:1:1. Enrollment was carried out by Principal investigator

Ethics Committee Approval was obtained from the Institutional Ethics committee of Amrita Institute of Medical sciences dated Aug 8 2011. The trial was registered with the CTRI/2011/12/002227.

### Intervention

#### Yoga Intervention

Instructor driven yoga sessions were conducted for 60 min on two days a week. Yoga sessions were conducted by the yoga instructor who had a diploma in yoga and Naturopathy and was assisted by two trained persons with masters in medico-social work (MSW). On the other days the women were instructed to practice at home and maintain a daily log. The 60 min sessions consisted of 25 min-Surya namaskara-12 steps, 5–7 min-Deep relaxation-Muscle relaxation technique, 15 min Asana or yoga postures. The postures consisted of Pavanamuktasana in the supine position, Bhujangasana and Shalabhasana in the prone position, Ardhamatsyaendrasana in the sitting position as recommended by yoga experts for diabetes [17]. This was followed by 15 min of pranayama. A record of the food eaten, drugs consumed and exercise particulars were also maintained for two days a week considered to be representative of the entire week. This was reviewed every month. On the basis of this record the dietician determined adherence to diet based on the approximate calorie consumption in relation to the type of work done (sedentary/moderate/heavy work).

If any of the study participants were found to have hypoglycaemia (Random plasma glucose (RPG) < 70 g/dl) in the monthly review with or without symptoms the oral drugs was reduced by 20–50%.

#### Peer support intervention

Three Peer mentors were identified from the community and trained. The criteria for eligibility was; having had type two Diabetes for at least one year with a RPG ≤250 mg/dl in the last reading, judged by the investigation team to be generally adherent to treatment and behaviour change regime, capacity and commitment to undergo the training required, an understanding of patients confidentiality, undertaking to liaise with the concerned doctor if unanticipated problems arose during the course of their peer support activity. The peer mentors provided support to the study participants in a ratio of 1:14. Peer Mentors underwent a two day training programme consisting of a physician who explained aetiology of diabetes, changes taking place in the body due to Diabetes, complications due to poor glycaemic control and an outline on the drugs used and its mechanism of action and the synergies with physical activity. The nutrition specialist explained all the nutritional and dietary aspects of diabetes; psychologist trained the peer mentors on communication skills, empathy, confidentiality. A training manual was prepared for the peer mentors based on the peers for progress handbook and handed over to the mentors for future reference.

Peer support meetings: Each peer mentor would visit 13–14 women with diabetes. A face to face meeting with the woman with diabetes in a week for about 45–60 min on assistance in applying disease management or prevention plans in daily life, providing emotional and social support and pro active flexible ongoing support. This was followed up by a telephone call in the same week. A monthly review of the activities was also undertaken by the principal investigator. During the first visit, the peer mentor collected the treatment details including drugs, diet and physical activity. In the follow up visits the peer mentor advised and monitored the woman regarding diet, exercise, timely consumption of drugs, emotional stress, symptoms, foot care etc. In the third month during the last visit the peer mentor conducted a final assessment regarding the entire process, its acceptability, difficulties and usefulness to the woman with diabetes. The woman with diabetes in the peer support group was also given a diary to record the visit, advice of the peer mentor and the changes brought about.

The control group was given the usual standard of care including continuing oral hypoglycaemic drugs, advise on diabetic diet and exercise for at least 10 min a day to a level of 150 min/week. All the patients were reviewed monthly and necessary care given. A pretested, semi structured questionnaire was used to collect socio demographic and other information.

### Outcome measures

The primary outcomes studied were fasting plasma glucose, HbA1c, quality of life and pharmacological adherence. The effect on quality of life will be discussed elsewhere. Adherence to drugs was measured by Morisky Medication adherence scale (MMAS–8) [18] in the local language, Malayalam and it was scored according to guidelines with a maximum score of 8. Fasting plasma glucose was measured by Hexokinase method, HbA1c by the High performance liquid chromatography method and cholesterol oxidase-peroxidase method for cholesterol. The secondary outcomes studied were BMI and waist hip ratio (WHR), blood pressure and total cholesterol (the last two were added later as funds became available). Anthropometric measurements like weight, height, waist and hip circumference were recorded by standard methods [19] at the beginning before randomisation and at the end of three months at a post treatment visit. Those who took the measurements were not aware of the group to which the patient belonged.

To understand the process, feedback was collected from both the intervention groups. Yoga group’s attendance was monitored and more than 80% of attendance in instructor classes was considered as regular. Dietary adherence was operationalized by recording the food eaten on the day previous to the yoga class. The total calorie consumed was calculated by the dietician according to the nature of work and was deemed to be adherent or non adherent.

Statistical methods: Mean and Standard Deviation (SD) were computed for all the measurable study variables for each intervention group. To test significance of the differences in mean values of the study variables, from basal to the follow-up period; within each group, paired t test was applied and among the three groups, one-way Analysis of Variance was applied. In case of statistical significance, multiple comparison test was applied to identify significantly different pairs of groups.

## Results

A total of 180 women with type two diabetes were screened and 56 women were excluded due to various reasons (Fig 1). Thus, 124 women were randomized into three groups. The study population comprised of peri-menopausal women with a mean age of 51.9 (7.3) and the mean duration of diabetes was 5.4 (2.8) yrs.

The socio-demographic characteristics, biochemical and anthropometric measurements of the population at baseline like mean age, mean duration of diabetes, BMI, WHR, FPG and HbA1c showed no significant differences between the three groups indicating that they are comparable (Insert Table 1).

**Table 1:** Comparison of baseline values in Yoga, Peer support and control groups

	Yoga			Peer support			Control			
	*n*	Mean	SD	*n*	Mean	SD	*n*	Mean	SD	*p*
Age (in yrs)	35	51.97	7.40	33	51.92	8.32	38	51.92	6.57	0.99
Per capita Income (in INR)	35	986.57	1992.32	33	715.03	1356.35	38	1602.13	4927.7	0.49
^a^FPG (mg/dl)	35	163.27	46.56	31	177.99	70.39	38	186.69	73.02	0.29
^b^HbA1c % (mmol/mol)	35	9.5(80)	1.65	31	9.4 (79)	1.62	38	9.6 (82)	1.85	0.89
Body mass index(kg/m2)	34	24.83	3.89	31	25.54	4.52	36	24.68	4.1	0.67
Waist Hip Ratio	34	0.97	0.05	32	0.97	0.04	37	0.9	0.05	0.42
^c^BP systolic (mm Hg)	34	134.76	20.63	32	128.69	18.07	38	124.63	15.7	0.06
BP diastolic (mm Hg)	34	84	11.43	32	83.5	9.5	38	79.63	7.64	0.11
Adherence	31	5.46	1.92	27	5.36	1.74	37	5.08	1.67	0.66
Total Cholesterol(mg/dl)	35	220.65	53.29	31	234.98	38.23	38	216.63	63.39	0.34
Diabetes duration(yrs)	32	5.8	2.78	34	5.34	2.75	37	5.1	3.04	0.62

^a^ *FPG*: Fasting plasma glucose

^b^ *HbA1c*: Glycosylated Haemoglobin

^c^ *BP*: Blood pressure

The mean systolic and diastolic BP showed a significant decrease in the yoga group with a fall of 6.1 mm systolic (p = .08) and 3.1 mm diastolic blood pressure (*p* = .03). The glycaemic outcomes and other variables are as shown in table 2. (Insert Table 2) In, between the group comparison of the primary outcome of FPG and HbA1c there was a mean fall of FPG by 6.5 mg/dl in yoga group (CI 34.5 − −44) as compared to 5.9 mg/dl in peer (37.6 – −46.1) and 1.7 mg/dl in control (−31.6 – 28) though this was not significant. The differential fall in HbA1c was 0.3%(CI −0.85 – 0.34) in yoga; an increase in peer and control by 0.5%(CI −0.32,1.4) and 0.19%(CI–0.19,0.86) respectively was observed. Thus only yoga group showed a decrease in HbA1c, though not significant. Morisky’s adherence score showed an increase in all the three arms and the highest increase was seen in the control group at 0.7 (CI.09,1.4) though it was not significant.

**Table 2:** Within group analysis before and after the intervention

	Yoga				Peer				Control			
Variable	No	Mean	SD	*p*	no	Mean	SD	*p*	no	Mean	SD	*P*
^a^FPG(mg/dl) Pre	32	166	43.03	0.51	25	169.5	62.78	0.6	30	183.58	72.16	0.9
Post	32	159.5	44.08		25	163.54	69.72		30	181.84	71.77	
^b^Hb A1C% Pre (mmol/mol)	32	9.6 (82)	1.62	0.39	25	9.3 (78)	1.6	0.15	30	9.4 (79)	1.71	0.5
Post	32	9.4 (79)	1.54		25	9.8 (84)	2.26		30	9.6 (81)	1.94	
Adherence Pre	30	5.55	1.88	0.1	21	5.42	1.67	0.58	29	5.03	1.79	0.02
Post	30	6.07	1.49		21	5.61	1.96		29	5.75	1.49	
Systolic^c^ BP(mmHg) Pre	32	133.81	20.91	0.02	26	127.07	19	0.55	30	126.06	16.17	0.34
Post	32	127.75	18.62		26	129.46	19.21		30	129.26	17.91	
DiastolicBP(mmHg) Pre	32	84.25	11.74	0.07	26	81.84	9.39	0.66	30	80.2	7.34	0.06
Post	32	81.12	9.18		26	81	10.6		30	83.3	8.5	
Total Cholesterol(mg/dl) Pre	32	218.87	48.81	0.34	25	233.19	40.93	0.32	30	211.37	36.42	0.09
Post	32	225.13	44.76		25	228.24	35.82		30	227.2	55.59	
Waist Hip Ratio Pre	32	0.96	0.045	0.34	27	0.97	0.04	0.02	30	0.98	0.051	0.002
Post	32	0.95	0.053		27	0.94	0.04		30	0.94	0.054	
Body Mass Index (kg/m^2^) Pre	32	24.74	3.97	0.09	31	25.54	4.52	0.05	35	24.86	4.07	0.01
Post	32	22.82	7.16		31	21.72	9.36		35	20.32	10.06	

^a^ *FPG*: Fasting plasma glucose

^b^HbA1c: Glycosylated Haemoglobin

^c^ *BP*: Blood pressure

Among the secondary outcomes; Yoga had brought about a significant differential decrease in diastolic blood pressure by 3 mmHg (CI–6.4, – 0.77; *p* = .035) as compared to a marginal decrease in peer and an increase in control group. Total cholesterol levels in the peer group decreased by 5 mg% (95% CI–15,5.1) compared to an increase in yoga and control groups by 6 mg% (95% CI-7.3–19.5) and 16 mg% (95% CI-2–34.3) respectively which was again not significant.

Anthropometric measures such as; BMI showed an increase in all three groups with the yoga group having the smallest increase, though this was not significant. The mean fall in waist hip ratio was marginal and almost the same in peer and control groups at .04 and 0.03. Though, there was an increase in hip circumference in all the three groups, it was significantly the least in the yoga group (.4) and the most in the peer group (4.3).On applying multiple comparison test, on the significant variables, the difference in the hip circumference of the peer and yoga group was found to be significant (95% CI 7.16,1.00; *p* = .005) and the difference in diastolic blood pressure between yoga and control group was found to be significant (95% CI.41, 12.1, *p* < .03) (Insert Tables 3 and 4).

**Table 3:** Between group comparisons of differences in biological parameters

	Yoga			Peer support			Control			
	Mean	SD	95%CI	Mean	SD	95%CI	Mean	SD	95%CI	*p*
Diff FPG	–6.5	56.42	–30.12,16	–5.9	105.7	–30.5,24.25	–1.73	74.6	31.6, 28	0.9
HbA1c	–0.3	1.6	–0.93,0.4	0.5	4.29	–0.32,1.4	0.19	1.5	0.19,0.86	0.2
Diff sys BP	–6	14.23	–10.3, 0.03	2.4	20.4	–6.2, 13.4	3.2	18.1	3.4,11	0.08
Diff dias BP	–3.1	9.5	–6.4,0.77	–0.84	9.7	–4.4, 4.6	3.1	8.9	0. 26,7.16	0.03
Diff adher	0.52	1.7	–0.25,0.9	0.2	1.8	–0.09,1.4	0.7	1.6	0.09,1.4	0.6
Diff^a^ BMI	–1.92	1.7	–1.1,0.2	–3.82	2.2	–0.82,0.18	–.2	–4.5	–0.65,0.21	0.65
Diff^b^ WHR	–0.01	.17	–.02,.013	–0.03	.18	–0.04,–0.002	–0.04	0.05	–.05,–.01	0.12
Diff^c^ TC	6.2	36.8	–6.4,22.9	–4.9	24.4	–16,7.5	15.8	49.5	–5.1,34.3	0.15
Diff Hip circumference	0.25	4.27	–1.8,1.5	4.3	6.09	–7.5,1.8	2.7	4.04	–4.4,–1.3	0.006

^a^ *FPG*: Fasting plasma glucose

^b^ *WHR*: Waist hip ratio

^c^ *TC*: Total Cholesterol

**Table 4:** Multiple comparison test to identify significantly different groups

**Diff BP diastolic**	***p***	**95% CI**
*Yoga & Control*	*0.03*	.41 to 12.1
Peer & Control	0.35	–2.1 to 10.1
Yoga and Peer	1	–8.3 to 3.7
**Diff Hip circumference**	***p***	**95% CI**
Yoga & Control	0.14	–.52 to 5.45
Peer and Control	0.62	–4.7 to 1.5
*Yoga & Peer*	*0.005*	1 to 7.1

### Process

The women in the Yoga group recorded daily yoga asanas at home apart from twice a week hourly sessions under the guidance of a yoga instructor. The women in the yoga group were undergoing on an average about 30 min’s of yoga classes per day. Three fourths of the women underwent yoga classes regularly, where attending 80% of the instructors classes was considered as regular. Dietary Adherence ranged from a minimum of 40% to a maximum of 80% with most of the women stating difficulties in adherence to diet and was based on assessment by a dietician in terms of approximate calorie intake based on self reports. Adherence was difficult as they were primarily cooking to the tastes and needs of their families, children and grandchildren. Other exercises if any were also recorded.

In the peer support group, for the first two months the three peer supporters made regular visits and had one to one discussion with the patient. On the visits, peer mentors could not regularly contact about 15% (5/32) of women due to various reasons such as an employment program of the Government for poverty eradication, non availability etc. Of these 20% (1/5) of the women could not be directly contacted over phone too due to frequent change in phone numbers. In the third month one of the peer mentors dropped out citing personal circumstances. Half of the women with Diabetes in this group were not enthusiastic about the idea of peer support at the beginning, though towards the end of the program 94% (30/32) of the members reported that it was useful.

## Discussion

There has been a recent interest in yoga and peer support as low cost adjunctive therapies to diabetes. This study is unique in that it has been done among peri-menopausal women who are a challenging group, due to competing domestic concerns; and limited access to health care for various reasons including work burden [9].

Over all, the results of our study, which assessed the effect of Yoga and Peer Support on glycaemic outcomes of peri-menopausal women with diabetes, are essentially neutral. However, some interesting findings and trends do emerge. Yoga helped in reducing the diastolic blood pressures significantly, systolic blood pressure and there was a trend towards improvement in the blood glucose levels and body mass index.

FPG showed a declining trend in both peer and yoga group by 5.9–6.5 mg/dl in comparison to control and HbA1c fell by 0.3% only in the yoga group. Other yoga studies have demonstrated a fall in FPG ranging from 20–41 mg/dl [20–22] and HbA1c by 1.2% [21]. An intensive in-patient study among 149 patients for 40 days also showed that with a FPG < 140 mg/dl, hyper glycaemia could be controlled by yoga alone [12]. A rise in Insulin levels [22] accompanied by a fall in anthropometric measures like waist hip ratio suggesting a redistribution of fat and glucose utilization has also been reported. Though, the above mentioned studies among adults show a decrease in FPG,HbA1c; the extent of the decrease is debatable in the absence of a control group or comparison with a control group in some [12, 20–22] and small study groups [20–22] in others. In this study there are clear trends of decline in the yoga group, but, perhaps a longer time period is required for the effects to manifest in this challenging study group. The findings of our study are also in contrast to the conclusion of a systematic review report that RCT on yoga in India are more likely to have positive outcomes [23] in terms of glycaemic outcomes. A significant decline in diastolic blood pressure by 3 mmHg and a trend in decline of systolic blood pressure by 6 mmHg was observed indicating that yoga could also help in the successful management of co-morbidities like Hypertension. Other studies have also shown decline in blood pressure [16, 24] ranging between 4–9 mmHg. A surprising effect observed was the improvement in the body mass index in the control group. This could be related to their better socioeconomic status at baseline (though this was not statistically significant).

Studies in countries outside India give mixed reports with some reporting a significant improvement in HbA1c, FPG, lipid profiles [25, 26] whereas another exploratory study in UK showed only a marginal decline of 0.2% in HbA1c [27].

In peer studies all over the world, a Mexican American study showed a 1.7% decline in HbA1c by the 4^th^ month of study [28],a reciprocal peer support study showed a decrease in HbA1c [29] compared to others which either showed no effect [30] or only found it feasible with no improvement in biophysical outcomes [31]. Though this study showed a fall in FPG, there was no effect on HbA1c. The peer group also demonstrated a fall in total cholesterol levels by 5 mg/dl in comparison to the control group which showed an increase by 15 mg/dl. The process also indicated that it was feasible to carry out both the interventions though the adherence to the study could have been improved by compensating the money spent on travel and loss due to wages. The sustainability of peer mentoring was critical to the peer intervention, it may be necessary to look at other alternatives such as baseline community health workers to carry out the peer mentoring. Transcending the gender hierarchy is also a formidable challenge [32].

The study was limited by the fact that this is generalisable only to women in the peri-menopausal group. There was loss to follow up too at an average of 18%. It was feasible to conduct the study within the resource constraints as peer mentors and yoga instructor voluntered their time and were compensated only for their travel expenses. The participants also bore the direct and indirect expenses on the days of their visit to the health centre such as travel and loss of wages. Both yoga and peer support are innovative applications that have a potential to be used as adjuncts.

## Conclusion

A longer period of follow up is necessary to evaluate the precise contribution of yoga and peer support to glycaemic control though the yoga group demonstrated a significant decrease in diastolic blood pressure. Notably, control group was also strongly supported by conventional diabetes education and over and above that, the incremental effect of yoga and Peer support was minor, and not profound. We call for longer term studies to ascertain the benefits shown by our pilot study.

## Abbreviations

BMI: Body mass index
DM: Diabetes Mellitus
FPG: Fasting plasma glucose
HbA1c: Glycosylated haemoglobin
MMAS: Morisky medication adherence scale
RPG: Random plasma glucose
WHR: Waist hip ratio
